# Editorial: Granger causality and information transfer in physiological systems: basic research and applications

**DOI:** 10.3389/fnetp.2023.1284256

**Published:** 2023-10-13

**Authors:** Sonia Charleston-Villalobos, Michal Javorka, Luca Faes, Andreas Voss

**Affiliations:** ^1^ Department of Electrical Engineering, Metropolitan Autonomous University, Mexico City, Mexico; ^2^ Jessenius Faculty of Medicine, Comenius University, Martin, Slovakia; ^3^ Department of Engineering, University of Palermo, Palermo, Italy; ^4^ Institute for Biomedical Engineering and Computer Science, Ilmenau University of Technology, Ilmenau, Germany

**Keywords:** Granger causality, information transfer, physiological systems, basic research, applications, network physiology

The concept of causality provides a theoretical framework to gain insights into the mechanisms underlying driver-response relationships in coupled systems by estimating the involved subsystems’ directed interaction. In recent years, this approach has become fundamental to investigating coupled dynamical systems in several fields, including physiology, physics, and economics, among others (Bressler and Seth, 2011). In this context, a class of information-theoretic methods has been proposed for causality estimation of time series, starting from the fundamental definition of Granger causality (GC) based on multivariate linear autoregressive models (Barrett et al., 2010) and extending to non-linear and non-parametric information transfer approaches (Faes et al., 2011). In the field of network physiology, causality analysis has become a reference tool (Porta and Faes, 2016; Ivanov, 2021) as it has allowed studying the dynamics of brain connectivity, interactions of the brain with other organ systems, as well as the interactions between pairs of physiological systems such as the cardiovascular, cardiorespiratory, and cerebrovascular ones (Porta and Faes, 2016; Khandoker et al., 2019).

The present Research Topic highlights advances in causality analysis applied to bi- and multivariate physiological and simulated time series. The Research Topic has attracted nine high-quality papers involving novel methodologies, revisiting established approaches, and presenting innovative applications.

The methodological paper from Shao et al. introduced a novel measure of causal influence strength in the context of recurrent transient neural events—the relative dynamic causal strength (rDCS). This measure was compared to previously used tools and verified by simulated and experimentally recorded neurophysiological data. The significance of this approach to studying brain activity mechanisms was further discussed.

A modified, corrected measure of the mutual information rate (cMIR) was developed by Mijatovic et al. for the study of bivariate point processes. The functionality of the measure was first demonstrated in various simulation studies, showing its good performance in terms of bias reduction, and then in real point process data represented by heartbeat times and arrival times of the sphygmic wave at the body periphery. In the application, a significant coupling between the two processes could be demonstrated in healthy subjects studied across different experimental conditions. The statistically significant changes in cMIR observed during physiological stress suggested that this index may reflect neuroautonomic modulation of heartbeat and vascular dynamics.

As a decision aid for interaction analyses, Günther et al. compared strengths and weaknesses of the GC approach and the Bivariate Phase Rectified Signal Averaging (BPRSA). Among other results, they found that BPRSA requires more data or stronger interactions than GC and that the latter, unlike BPRSA, can detect direct causal relationships from indirect relationships. In contrast to this, BPRSA is suitable for the analysis of non-stationary data. The suitability of GC for the coupling analysis of causal networks in subjects with and without sleep apnea (analysis of heart rate, respiratory rate, and EEG alpha amplitude) was successfully demonstrated.

Baccalà and Sameshima delved into the methodological aspects of the analysis of GC, particularly focusing on the assumptions underlying the computation of GC measures in both time and frequency domains. Importantly, they show that using the very popular linear vector autoregressive model representation of the observed set of time series is not a mandatory requirement for computing GC but is rather a convenient representation that can be substituted by other means of spectral factorization of the spectral density matrix into minimum phase factors.

Important methodological aspects in the computation of GC are also addressed by Koutlis et al., who challenged the notion that the structure of neural connectivity underlying multichannel electroencephalographic signals can be better inferred by analyzing the cortical source time series obtained through inverse source reconstruction. Using both simulated and real signals measured from epileptic subjects, they showed that causality networks constructed at the sensor and source levels differ significantly, and the former can yield better discriminative ability of network topological indices.

Froese et al. employed GC and other statistical methods applied to autonomic response variables such as heart rate and blood pressure variability to investigate the relationship between such variables and cerebrovascular reactivity in patients with moderate to severe traumatic brain injury. The study showed that the sympathetic autonomic response, monitored via spectral indexes and baroreflex sensitivity, is closely linked to impaired cerebrovascular reactivity.

Incorporating measures of physiological interaction in classification tasks is gaining importance due to consideration of the relevant physiological information. In that sense, Difrancesco et al. set out to improve the performance of machine learning models, specifically support vector machine classification algorithms (SVM), by including cardiorespiratory interactions to identify and classify acute toxicity effects of two chemicals on guinea pigs, an opioid and a nerve agent. For this purpose, by continuously monitoring the ECG and respiration signals through wearable sensors, the F-statistics of bivariate prediction models between respiration features, as the tidal volume, and ECG morphology characteristics, as the ST elevation, were fed to an SVM. Although chemicals affected cardiorespiratory interactions differently, they did not improve SVM performance since respiratory features were the most important for the classification task.

In the search for new ways to measure cerebral blood flow (CBF) in humans non-invasively and continuously, González et al. proposed the rheoencephalograph (REG) signal as a CBF surrogate to provide patient monitoring in surgeries. Furthermore, the authors addressed the estimation of bivariate GC to understand better the anesthetics’ effects on brain hemodynamics. GC was estimated between REG’s features, linear and non-linear, and global hemodynamics by HR or MAP, as well as EEG-based parameters related to the depth of anesthesia. Results pointed out that REG effectively contains information on CBF, and the most frequent causal interaction occurred from CBF_REG_ to the different EEG spectral density bands but not in the opposite direction.

In a clinicall-oriented research study, Santiago-Fuentes et al. applied univariate and causal bivariate analyses on cardiorespiratory signals obtained from patients with idiopathic pulmonary fibrosis (IPF) before and during oxygen supplementation to characterize cardiovascular control and cardiorespiratory interactions. In IPF patients, a shift of the sympathovagal balance towards sympathetic dominance was observed, accompanied by a decreased cardiorespiratory interaction, increased blood pressure variability, and decreased baroreflex function during oxygen supplementation. These results point towards a persistence of autonomic control impairment despite oxygen administration.

The papers of this Research Topic consider fundamental aspects of causality estimation and include novel proposals. Also, most of the contributions showed the power of the causality framework for dissecting intricate directed interactions from physiological networks studied in diverse physiological and pathological scenarios. These efforts are hoped to increase the impact and importance of causality estimation in clinical settings, among others.
